# Whole genome amplification and real-time PCR in forensic casework

**DOI:** 10.1186/1471-2164-10-159

**Published:** 2009-04-14

**Authors:** Emiliano Giardina, Ilenia Pietrangeli, Claudia Martone, Stefania Zampatti, Patrizio Marsala, Luciano Gabriele, Omero Ricci, Gianluca Solla, Paola Asili, Giovanni Arcudi, Aldo Spinella, Giuseppe Novelli

**Affiliations:** 1 Centre of Excellence for Genomic Risk Assessment in Multifactorial and Complex Diseases, School of Medicine, Tor Vergata University of Rome, Rome, Italy; 2 Direzione Centrale Anticrimine, Servizio di Polizia Scientifica, Rome, Italy; 3 Department of Public Health – Institute of Forensic Medicine, Faculty of Medicine, Tor Vergata University of Rome, Rome, Italy; 4 Division of Cardiovascular Medicine, Department of Medicine, University of Arkansas for Medical Sciences, Little Rock, AR 72205, USA

## Abstract

**Background:**

WGA (Whole Genome Amplification) in forensic genetics can eliminate the technical limitations arising from low amounts of genomic DNA (gDNA). However, it has not been used to date because any amplification bias generated may complicate the interpretation of results. Our aim in this paper was to assess the applicability of MDA to forensic SNP genotyping by performing a comparative analysis of genomic and amplified DNA samples. A 26-SNPs TaqMan panel specifically designed for low copy number (LCN) and/or severely degraded genomic DNA was typed on 100 genomic as well as amplified DNA samples.

**Results:**

Aliquots containing 1, 0.1 and 0.01 ng each of 100 DNA samples were typed for a 26-SNPs panel. Similar aliquots of the same DNA samples underwent multiple displacement amplification (MDA) before being typed for the same panel. Genomic DNA samples showed 0% PCR failure rate for all three dilutions, whilst the PCR failure rate of the amplified DNA samples was 0% for the 1 ng and 0.1 ng dilutions and 0.077% for the 0.01 ng dilution. The genotyping results of both the amplified and genomic DNA samples were also compared with reference genotypes of the same samples obtained by direct sequencing. The genomic DNA samples showed genotype concordance rates of 100% for all three dilutions while the concordance rates of the amplified DNA samples were 100% for the 1 ng and 0.1 ng dilutions and 99.923% for the 0.01 ng dilution. Moreover, ten artificially-degraded DNA samples, which gave no results when analyzed by current forensic methods, were also amplified by MDA and genotyped with 100% concordance.

**Conclusion:**

We investigated the suitability of MDA material for forensic SNP typing. Comparative analysis of amplified and genomic DNA samples showed that a large number of SNPs could be accurately typed starting from just 0.01 ng of template. We found that the MDA genotyping call and accuracy rates were only slightly lower than those for genomic DNA. Indeed, when 10 pg of input DNA was used in MDA, we obtained 99.923% concordance, indicating a genotyping error rate of 1/1299 (7.7 × 10^-4^). This is quite similar to the genotyping error rate of STRs used in current forensic analysis. Such efficiency and accuracy of SNP typing of amplified DNA suggest that MDA can also generate large amounts of genome-equivalent DNA from a minimal amount of input DNA. These results show for the first time that MDA material is suitable for SNP-based forensic protocols and in general when samples fail to give interpretable STR results.

## Background

Data on human genetic variations are being generated and used to improve understanding of human origins, individual susceptibility to illness, the causes of disease and the genetic determinants of responses to drugs. The same kinds of data that are used to analyze genetic differences among humans for medical purposes can be also used in courts of law to determine identity. Forensic genetics is the branch of genetics that, through DNA analysis and comparison, helps to resolve legal problems such as paternity tests, establishing identity in criminal cases where biological evidence is found at crime scenes, inheritance matters, identification of victims of mass disasters, and identification of missing persons from human remains [1,2]. In forensic genetics, DNA samples are analyzed by comparing DNA sequences that are unique to each individual. Although more than 99.5% of the genome is the same throughout the human population, variations in DNA sequence called polymorphisms can be used both to differentiate and to correlate individuals. Short Tandem Repeats (STRs) are the polymorphisms most commonly used as markers by forensic scientists. STRs consist of repetitive units of DNA that are 2–6 bp in length. The number of repeats in STR markers differs markedly among individuals, which make them effective for human identification purposes. Currently, different optimized multiplex assays are utilized to analyze multiple independent STR loci located on different chromosomes. The combination of alleles typed in each locus provides an exclusive genetic profile with a high power of discrimination for each individual, i.e. the probability of being able to discriminate between two people chosen at random from a given population [3].

However, a forensic laboratory often has to deal with DNA samples that are less than ideal. The technical capacity to analyze samples expected to contain very few cells is often limited by the quality and quantity of DNA. The main limitation of existing forensic DNA protocols lies in the minimum size and/or amount of DNA fragments that can be typed by a capillary electrophoresis assay (ranging from 100–400 bases in length and from 500–1250 pg) [4]. Sometimes forensic samples may contain only small amounts of genomic DNA (less than 100 pg), referred to as LCN DNA (low copy number DNA), and/or may also be degraded into fragments smaller than 100 bp, resulting in failure of amplification of the STR loci and an incomplete DNA profile with lower discriminatory power [5]. To overcome this problem, several protocols for whole genome amplification (WGA) have been developed. The aim of WGA is to amplify a limited DNA sample non-specifically in order to generate a new sample that is indistinguishable from the original but has a higher DNA concentration. This method was specifically designed to increase the quantities of DNA in clinically relevant samples such as single cell analysis, pre-implantation genetic diagnosis and forensic typing [6]. However, the application of WGA methods to forensic casework is demanding because of technical artefacts that are particularly relevant when STR markers are analyzed. Such artefacts include contamination, PCR failure, preferential allele amplification, the complete absence of one allele (allele drop-out, ADO) in heterozygous loci and the non-specific generation of extra alleles (allele drop-in) [7,8]. Recently, an isothermal WGA method was introduced using bacteriophage Φ29 DNA polymerase and random hexamer primers [9,10]. This method, termed MDA (Multiple Displacement Amplification), takes advantage of the bacteriophage Φ29 DNA polymerase, a proofreading enzyme with high fidelity and potent strand displacement activities. MDA is based on the isothermal strand displacement mechanism and leads to hyperbranching amplification of both circular and linear DNA templates [10]. Its advantages include limited bias in the amplification of loci (maximally 6-fold compared to 10^3^- to 10^6^-fold in PCR-based methods), higher average length of amplification products (12 kb compared to 100–1000 bp in PCR-based methods), lower error rate (<10^-6 ^compared to 3 × 10^-4 ^to 10^-5 ^for alternative methods) and higher DNA yield [11]. Recent data have shown that the distance to telomere predicts failure only in WGA samples while percentage-GC is positively correlated with PCR failure [12,13]. MDA has been recognized as the most effective WGA method but its applicability to forensic casework has not yet been considered because technical artefacts have not been eliminated [14,15]. Significantly more alleles are revealed when STRs are analyzed by application of MDA to LCN samples than to non-amplified control samples although false allele generation (allele drop-in) was reported. The cause of such artifacts are not yet completely explained although it appears more frequent in STRs analysis [7,16]. Moreover STR analysis in MDA samples also shows an increased number and height of stutter peaks together with a high level of allelic imbalance, affecting the interpretation of results, especially in cases where two or more individuals may have contributed to the DNA evidence [16,17]. Stutter peaks result from the PCR process when STR loci are copied by a DNA polymerase. Because stutter products are the same size as actual allele PCR products, it can be challenging to determine whether a small peak is a real allele from a minor contributor or a stutter product of an adjacent allele especially when the height of the stutter is higher than that normally expected [18].

Moreover, comparative analysis of genomic and MDA samples starting from 6 ng of DNA reported only a 90–95% concordance rate for STRs [19,20]. The amount of template gDNA required for the MDA reaction is also a critical determinant of genotyping performance because it is generally unacceptable for forensic purposes. Thus, since current forensic profiling is based on STRs and the concordance rate with LCN DNA has not proved acceptable for forensic profiling, MDA is not regarded as valuable for typical forensic casework. However, better concordance rates were observed when MDA was applied to SNPs. Until now, the STR concordance rate has been worse for amplified than for genomic DNA, even with an input of 200 ng of gDNA into the MDA reaction; whereas optimal TaqMan^® ^SNP genotyping is expected for MDA DNA inputs greater than 4 ng [21]. With less than 5 ng genomic DNA in the MDA reaction, the amplification bias and the number of genotyping errors increases [17,22]. Similar technical limitations were also reported when MDA was considered for pre-implation genetic diagnosis, with up to 32% ADO for single-cell samples [6].

Recently, we developed and validated an SNP panel for human identification, rigorously selected to have very constant frequencies throughout the population tested and showing high specificity for the human genome [5]. The average size of our amplicons (77 bp) is much less than current forensic markers, making them more sensitive for LCN DNA and less prone to PCR bias.

Our aim in this work was to assess the applicability of MDA to forensic SNP genotyping by performing a comparative analysis of genomic and MDA DNA samples. A 26-SNPs TaqMan panel specifically designed for LCN and/or severely degraded genomic DNA was typed on 100 genomic as well as MDA DNA samples. We have confirmed the utility of a new TaqMan SNP typing reaction mix for typing degraded DNA samples. Also, amplification of experimentally degraded DNA using MDA, prior to TaqMan assays, eliminated a 4% failure rate for genotyping calls that occurred for unamplified genomic DNA samples. We have optimised the performance of TaqMan SNP typing of amplified LCN DNA and also tested the ability of MDA to type ten artificially-degraded DNA samples, which gave no results when analyzed by current forensic methods.

## Results

The aim of this work was to assess the applicability of MDA to forensic SNP genotyping. We explored the possibility of generating, from a minimal amount of input DNA (≤ 1 ng) in the MDA reaction, genome-equivalent DNA that can be efficiently and accurately typed for a large number of SNPs. We used a 26-SNPs TaqMan panel recently developed for LCN and/or severely-degraded genomic DNA.

We first assessed the performance of multiple displacement amplification (MDA) in terms of genotype concordance between gDNA (50 ng) used as reference genotype, genomic and amplified LCN DNA (≤ 1 ng). Initially, a human-specific real-time PCR assay, a Quantifiler™ Human DNA Quantification Kit (Applied Biosystems, Foster City, CA), was used to quantify the genomic DNA in 100 selected samples. All the samples were re-sequenced to assess the genotype at each locus tested. We then prepared serial dilutions of the DNA to obtain concentrations ranging from 1 to 0.01 ng/μl. MDA was performed on 1 μl of the three serial dilutions (1 ng, 0.1 ng, 0.01 ng) for each sample. The MDA products were quantified using the Quantifiler™ Human DNA Quantification Kit; 14.5, 1.5 and 0.27 μg of DNA were produced from the 1 ng, 0.1 ng and 0.01 ng dilutions, respectively. These quantities are only indicative, because Quantifiler amplifies a small fraction of the hTERT gene, which is located adjacent to the 5p telomere, and previous studies have shown that telomeric regions may be under-represented [16]. We also quantified the MDA products using Nanodrop spectrophotometer. The mean final yields of MDA obtained from the 1 ng, 0.1 ng and 0.01 ng dilutions were 19.5 μg, 8.5 μg and 6.5 μg, respectively. As previously reported [23] a considerable part of DNA obtained after MDA was a nonspecific DNA products. The RNAse P real-time assay was performed to estimate the amount of specific DNA obtained after MDA (10.0 μg, 2.6 μg and 0.3 μg for each dilutions respectively). TaqMan assays were performed on 1 μl of MDA DNA and gDNA. To evaluate the genotype concordance, all the TaqMan assay results from serial dilutions of gDNA and MDA DNA (1 ng, 0.1 ng and 0.01 ng) were compared to genotypes previously confirmed by direct re-sequencing of the same samples (50 ng). In parallel, TaqMan SNP assay results were examined on the same dilutions of gDNA as technical controls (Additional file 1). Optimal genotype concordance (100%) was observed between the sequence and TaqMan. The TaqMan assay performed on 1 ng of genomic DNA revealed no failure of amplification (100% call rate) and optimum genotype concordance (Additional file 1). The TaqMan assay performed on an input of 1 ng in MDA revealed PCR failure for the marker rs11881170 (99.960% call rate) but perfect genotype concordance. When the TaqMan assay was performed on 0.1 ng of genomic DNA, 100% genotype concordance was confirmed but there was failure of amplification in two samples for the markers rs3130315 and rs10866988, in six samples for marker rs585070 and in four samples for rs873289, resulting in a call rate of 99.461%. The TaqMan assay performed on a 0.1 ng input in MDA revealed a single PCR failure for the same marker, rs11881170 (99.961% call rate) and a single genotype discrepancy for rs1981752 (concordance rate 99.961%). When the TaqMan assay was performed on 0.01 ng of genomic DNA, 100% genotype concordance was again confirmed but there were more failures of amplification: in four samples for marker rs3130315, in two for marker rs585070, in 14 for rs1506981, in four for rs478347 and in six for rs873289. The resulting average call rate was 98.846%. In contrast, the TaqMan assay performed on a 0.01 ng input in MDA revealed a non-absolute concordance rate (99.379%) with two genotype discrepancies observed for marker rs2278741, two for rs7740233, two for rs10866988, two for rs1533800, two for rs154659 and three for rs873289. It is nevertheless remarkable that more positive calls were observed with 0.01 ng input of MDA-DNA than with unamplified DNA at same dilution. In particular, 20 samples failed to be typed (99.230% call rate) compared to 30 PCR failures with unamplified DNA at 0.01 ng dilution.

In order to determine whether a different reaction mixture might improve both concordance and call rates, we tested a recently-released master mix, the TaqMan^® ^Genotyping Master Mix (Applied Biosystems). This is able to optimize the preferential binding of the allele-specific probe, providing better separation and clustering of alleles and consistently strong fluorescence signals. Better results were obtained by repeating the same serial experiments under identical conditions using this new reaction mix (Additional file 2). In particular, all three dilutions of genomic DNA gave 100% results for both concordance and call rates. Also, we observed 100% concordance and call rates for MDA inputs of 1 ng and 0.1 ng DNA and 99.923% concordance and call rates for an MDA input of 0.01 ng (Additional file 2). We failed to assess the correct genotype in two out of 2598 samples, resulting in a genotyping error rate of 7.7 × 10^-4^. The two genotype discrepancies observed in MDA were ADO (false homozygous), and we also failed to find evidence that the typing results correlated with the GC content and/or the distance to telomeres. We note that this rate of genotype discrepancy is much lower than those reported for current STR forensic protocols [23,24]. We also never observed positive amplification in negative controls.

On the basis of these findings we selected the TaqMan^® ^Genotyping Master Mix as the default reaction mixture for our panel and for the further experiments presented here.

### Artificially degraded samples

**Figure 1:**
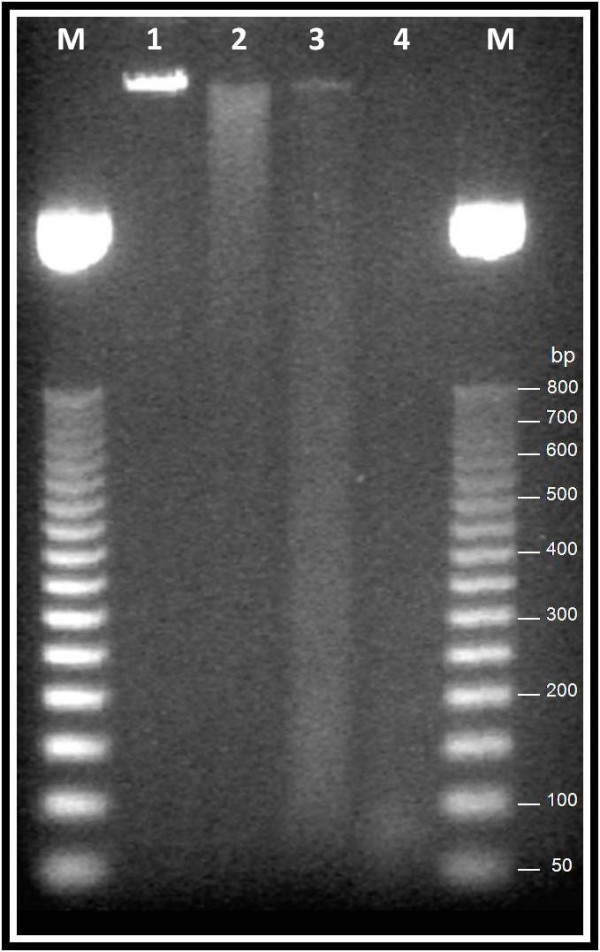
**Agarose gel (1.0%) analysis of digested DNA samples**. Lanes M are the size markers (50 bp ladder), lane 1 undigested genomic DNA, lanes 2–4 the digestion stopped after 30, 60 and 180 s, respectively. We decided to type samples digested for 180s, since they did not show evidence of partially-digested DNA fragments.

A specific digestion was performed to degrade 10 genomic DNA samples in such a way as to prevent the amplification of specific loci by current forensic technology (AMPFlSTR^® ^Identifiler^® ^kit). Control DNA was randomly degraded by DNase I (Promega Madison, WI, USA). The digestion reactions were stopped after 30, 60 and 180 s and DNase was removed by phenol-chloroform extraction. The average length of the degraded DNA (ranging from approximately 80 bp down to less than 50 bp for the digestion stopped after 180 s) was determined on an agarose gel (Figure 1). The digested DNA (180s) was subjected to MDA and quantified using a Quantifiler™ Human DNA Quantification Kit (Applied Biosystems, Foster City, CA), revealing an average amount of 2.41 ng. When we tried to amplify the 180 s-degraded genomic DNA samples with AMPFlSTR^® ^Identifiler^® ^kit (Applied Biosystems) under standard conditions [4], we failed to obtain positive results. The digested DNA (180s) was typed for the selected SNPs with or without prior MDA. The results of the TaqMan SNP genotypes obtained from gDNA and MDA DNA are shown in the Additional file 3. For degraded DNA samples, more than 4% of genotyping assays failed without a pre-amplification step using MDA. All of the samples could be genotyped following the MDA step.

## Discussion

In this work, we wished to investigate whether real-time TaqMan assays validated for use with low copy number (1 ng, 0.1 ng and 0.01 ng) or severely degraded genomic DNA might be of value for optimizing the performance of genotyping of MDA DNA.

To address this issue, we tested two independent protocols using our recently-developed forensic panel of SNPs. The first protocol revealed dissimilar results when genomic and MDA DNA were analyzed. In particular, 14 and 30 genomic DNA samples were not amplified with 0.1 ng and 0.01 ng dilutions, respectively, but all genotypes were concordant with the controls. In contrast, only one, one and 20 MDA DNA samples failed to be amplified with the 1 ng, 0.1 ng and 0.01 ng input dilutions, respectively. However, one MDA DNA sample (for inputs of 1 ng and 0.1 ng) revealed a genotype discordant with the controls, and 36 MDA DNA samples (for input of 0.01 ng) gave results dissimilar to the sequenced controls.

All discordant genotypes were false homozygous samples resulting from the complete failure of amplification of one allele in MDA (allele dropout, ADO) [7]. We therefore decided to use a different master mix specifically designed to optimize the preferential binding of the allele-specific probe. We repeated the experiments and obtained better results. We observed 100% concordance and call rate for all dilutions of genomic DNA and for inputs of 1 ng and 0.1 ng in MDA. Furthermore, when inputs of 0.01 ng were analyzed, we observed a single PCR failure and only two samples gave results dissimilar to the controls. The resulting concordance and call rate were therefore increased to 99.923% for a 0.01 ng input in the MDA reaction, resulting in a probability of genotyping error of 7.7 × 10^-4^, quite similar to that reported for STRs. Such results suggest that in combination with extra-short TaqMan assays and the specifically-designed master mix, MDA gave optimal findings in terms of sensitivity, reproducibility and robustness of genotyping. Independent studies have suggested that replicate MDA reactions should be pooled prior to genotyping in order to overcome the frequency of ADO and/or genotyping errors [25]. Tzvetkov observed that the concordance between MDA and gDNA genotypes shows a small but significant improvement (0.5%) in pooled samples compared to single MDA. In our opinion, this strategy could be applied when sufficient DNA template is available.

Nevertheless, the comparison between genomic and amplified DNA showed that MDA does not improve our ability to type LCN DNA for single SNPs, as demonstrated by the better concordance and PCR failure rates in genomic than in MDA DNA. The genotype concordance and PCR failure rates were 100% and 99.923% in MDA DNA and genomic DNA respectively. However, it should be noted that MDA amplification produces large amounts of DNA, so it is possible to type many SNPs; in contrast, genomic DNA is often present in limited quantities so fewer SNPs can be typed. Finally, MDA allowed us to type a large number of SNPs starting from LCN DNA as template. Moreover, our experiments on artificially-degraded DNA provide evidence that MDA can enhance our capacity to type severely-degraded samples. In our experiments, nine samples failed to be amplified in genomic DNA but were successfully typed in the amplified DNA. We observed 100% concordance and call rate for all SNPs using MDA on degraded DNA. The quality of our results in typing digested DNA by MDA was surprising, although this has also been reported to a lesser extent for STR typing [16]. Even though we typed completely-digested DNA (the average length of the degraded DNA (180s) was 80 bp down to less than 50 bp), it is likely that a small number of remaining intact fragments of template were amplified by MDA. In our opinion, these results depend on the marker types, the amplicon sizes and the genotyping technique. The results confirm and extend previous comparative analyses of SNP and STR typing in degraded forensic DNA (without MDA), demonstrating that good markers for degraded DNA depend on a small amplicon size [26]. Here, very small amplicons (77 bp on average) have been typed by the most sensitive genotyping assay.

Finally, we recommend using MDA in order to overcome limitations of DNA quantity (when the amount of DNA is limited) or when only poor-quality DNA samples are available (severely-degraded samples, to increase the number of typed loci).

We would also point out that 0.26 ng of genomic DNA (0.01 ng for each locus) is needed to type all 21 autosomal SNPs of our panel and 5 extra SNPs [5,27] successfully. It is also noteworthy that under these conditions, even though Real-Time PCR cannot allow multiplexing of the PCR, the amount of DNA needed to type a panel of SNPs in singleplex is comparable to current forensic STR methods and is much less than is needed to type forensic SNPs, as reported elsewhere [27]. As a result, its use in forensic practice should be seriously considered when traditional forensic kits fail.

If the amount of DNA proves insufficient to type all SNPs independently, it will be possible to apply MDA even in LCN templates. In this case, only 0.01 ng of input DNA is needed to type a large number of SNPs (even more than the 26 reported here). This amount of DNA is much less than needed in the forensic protocols reported to date, so it represents a significant improvement in current forensic DNA protocols. As mentioned above, the possibility of a technical artefact (i.e. ADO) or a mis-genotyping following MDA with 0.01 ng of input DNA is 7.7 × 10^-4^. This is a lower error rate than that reported for the STRs used for forensic typing. Moreover, the mutation rate is higher in STRs than in SNPs: germline mutations at these STR loci lead to problems in the interpretation of genetic profile results.

In order to compare SNP typing between two or more samples, we can state – by analogy with STR analysis – that there is a match (biological compatibility) when there are no differences at genotypic level for any of the SNPs tested. In contrast, if SNP genotypes show differences, we can state that the samples considered originated from different sources (exclusion). Finally, if SNP genotypes show just a few differences (one or two), we can state that the data are inconclusive because the information is ambiguous or insufficient to support any conclusion, since genotyping errors or ADO events cannot be excluded. Our results suggest that the probability of two genotyping errors or ADOs in the same sample is 5.9 × 10^-7 ^when only 10 pg of DNA are analyzed. Although this probability is quite low, homozygous genotypes in LCN DNA should be used with caution to exclude a match (compatibility): in this case, additional heterozygous markers should also be considered.

## Conclusion

In conclusion these results provide evidence that MDA can be considered suitable for SNP-based forensic protocols and in general when samples fail to give interpretable STR results. The demonstrated potential of MDA, in contrast to all previous WGA methods, is particularly promising and might have broad applications and significant implications in the areas of forensics and genetic studies.

## Methods

### gDNA samples

Blood or saliva samples from 100 unrelated subjects were extracted using QIAamp DNA Blood Mini Kit (Qiagen Inc., Valencia, CA). All samples were quantified using a Quantifiler™ Human DNA Quantification Kit (Applied Biosystems) according with manufacturer's protocols [28]. The hundred samples were diluted to 1, 0.1 and 0.01 ng/μl with double-distilled water.

### Amplification and sequencing

PCR amplifications were carried out separately for each SNP using 1 μl of DNA in a 30 μl reaction volume containing final concentrations of 1× True Allele Master Mix (Applied Biosystems) and 0.2 μM of each primer (Invitrogen) plus water. Oligonucleotide sequences have been described elsewhere [5,28]. The amplicons were denatured at 95°C (12 min), put through 28 reaction cycles of 95°C for 30 s, 58–65°C for 40 s and 72°C for 40 s, purified enzymatically (1 U exonuclease I and 2 U alkaline phosphate) (Ambion, Austin, Texas, USA) and directly sequenced by cycle sequencing with a BigDye Terminator v3.1 Cycle Sequencing Kit (Applied Biosystems) using the same primers as for amplification. The sequencing reactions were carried out in a 20 μl final volume containing 2 μl BigDye Terminator RR Mix, 1 μl 5× Big Dye Sequencing Buffer, 10 pmol primer, 2–4 μl PCR product and water. After a first denaturation step at 96°C for 1 min, 28 cycles of 10 s at 96°C, 5 s at 50°C and 2 min at 60°C were performed. Each template was sequenced in both forward and reverse directions using the amplification primers. Sequencing reaction products were purified from the residual dye terminators using CentriSep columns (Princeton Separations, Adelphia, NY, USA). Electrophoretic separation was carried out on an ABI Prism 310 Genetic Analyzer.

### DNA degradation

Ten artificially-degraded DNA samples were prepared by DNAase I digestion (Ambion Inc, Austin, Texas, USA). A total of 1 μg of DNA was digested with 1 U of DNAase I at 37°C in a solution containing water to 60 μl final volume. The reactions were stopped after 30, 60 and 180 s, adding 20 μl of the solution at each time point to 2 μl of 20 mM ethylenediaminetetracetic acid (EDTA). DNase was removed by phenol-chloroform extraction. To evaluate the degradation of genomic DNA, 3 μl of the digestion products were separated on a 1% agarose gel (figure 1).

### Whole genome amplification

Multiple displacement amplification WGA (MDA) reactions were performed using a Φ29 DNA polymerase-based Repli-g Mini kit (Qiagen) for each LCN sample (1, 0.1 and 0.01 ng/μl) and for the degraded samples (180 s digestion time). A total of 1 μl of genomic DNA was incubated with D1 solution (containing Reconstituted Buffer DLB and nuclease-free water) and then with N1 solution (containing Stop Solutions and nuclease-free water) for 3 min and the products were added to 7.25 μl Repli-g Mini Reaction Buffer and 0.25 μl Φ29 DNA polymerase. The mixture was incubated at 30°C for 8 h followed by heat-inactivation at 65°C for 3 min.

WGA products were quantified using three different methods: the Quantifiler™ Human DNA Quantification Kit (Applied Biosystems), Real-Time RNase P gene dosage assay (Applied Biosystems), and Nanodrop ND-1000 spectrophotometer (Nanodrop Technologies, Wilmington, DE, USA) at 260 nm. Quantitative Real-Time PCR reactions for RNase P gene was carry out in a final volume of 25 μl containing 12.5 μl of 1× TaqMan^® ^Expression PCR Master Mix (Applied Biosystems), 1.25 μl TaqMan^® ^RNase P Control Reagents (Applied Biosystems) and water. For the absolute quantification, calibration curve was generate with a serial diluitions of gDNA sample of known concentration ranging from 0.01 to 100 ng/μl.

### SNP genotyping of LCN

Serial dilutions of gDNA (1 ng, 0.1 ng and 0.01 ng) and 1 μl of each of the three MDA reactions (starting from DNA inputs of 1 ng, 0.1 ng and 0.01 ng) were used as templates for genotyping the 26 SNPs using the TaqMan^® ^Assay, to evaluate the allele calling and concordance rates. The list of SNPs together with their relative chromosome locations, PCR primer sequences and relative TaqMan^® ^probes were as described [5,28]. The reaction mixture for all SNP assays was as follows: 6.25 μl 1× TaqMan^® ^Universal Master Mix, 0.25 μl TaqMan Genotyping Assay and water to a final volume of 12.5 μl. Cycle conditions were 50°C for 2 min, 95°C for 10 min, 50 cycles of 95°C for 30 s and 60°C for 1 min, performed in a 96-well optical plate. Each plate contained three positive controls (samples previously confirmed by direct sequencing as heterozygous and both homozygous types) and two negative controls. gDNA and all MDA DNA were amplified simultaneously in duplicate, and genotypes were determined.

For the MDA DNA reaction, the new TaqMan^® ^Genotyping Master Mix was used. The reaction mixture was: 12.5 μl 1× TaqMan^® ^Genotyping Master Mix, 0.25 μl TaqMan^® ^Genotyping Assay and water to a final volume of 25 μl.

Fluorescence was detected using an ABI 7500 Sequence Detection System and genotypes were manually scored using Sequence Detection Software 2.0 (Applied Biosystems).

The SNP genotype call rate was calculated by dividing the number of correct amplification genotypes by the number of attempted genotypes; the SNP genotype concordance rate was calculated by dividing the number of concordant genotypes by the number of completed genotypes. The MDA DNA concordance rate was defined as the number of instances in which an MDA DNA SNP genotype differed from the scored gDNA SNP genotype.

### SNP genotyping assay of degraded samples

Aliquots of 1 μl of the 10 artificially-degraded DNA samples (180 s degradation time with DNAase I) and 1 μl of the same samples after the MDA reaction, together with 1 μl of genomic DNA, were tested for genotyping of the 26 SNPs on ABI 7500 using: 12.5 μl 1× TaqMan^® ^Genotyping Master Mix, 0.25 μl TaqMan^® ^Genotyping Assay, and water to a final volume of 25 μl. Artificially-degraded DNA, artificially-degraded MDA DNA and genomic DNA were amplified simultaneously in duplicate for each SNP analyzed.

### STR analysis

Artificially-degraded DNA (180 s degradation time with DNAase I) was used as template for the AmpFlSTR^® ^Identifiler^® ^Assay (Applied Biosystems). The AmpFlSTR^® ^Identifiler^® ^PCR Amplification Kit was used to amplify 15 STR loci simultaneously: D16S539, D2S1338, D19S433, D13S317, D8S1179, vWA, D21S11, D7S820, CSF1PO, D3S1358, THO1, D18S51, D5S818, FGA, TPOX and AMEL for gender determination. Amplification was carried out as previously described [4]. The total volume of each reaction was 25 μl. The PCR amplification was carried out in a 9700 Gene Amp PCR System Thermal Cycler (Applied Biosystems) according to the manufacturer's recommendations. Electrophoresis of the amplification products was performed on an ABI PRISM 310 Genetic Analyzer. The raw data were compiled and analyzed using accessory software GeneMapper™ 3.2 (Applied Biosystems).

## Authors' contributions

EG and IP conceived of the study and participated in its design and in the interpretation of the data. CM carried out the molecular genetic studies and statistical analysis. SZ, PM, LG, OR, GS and PA carried out the DNA extraction and sample quantification. GA, AS and GN reviewed the manuscript critically for important intellectual contents and coordinated the research group. All authors read and approved the final manuscript.

## Supplementary Material

Additional file 1 **Values of calls, concordant genotypes, concordance rate, call rate and genotype concordance in genomic and amplified DNA with TaqMan^® ^Universal Master Mix**. The genotyping results for each SNP are given for dilutions of 1 ng, 0.1 ng and 0.01 ng (first, second and third row respectively). Genotypes derived from direct sequencing were used as reference for determining concordance. Click here for file

Additional file 2 **Values of calls, concordant genotypes, concordance rate, call rate and genotype concordance in genomic and amplified DNA with TaqMan^® ^Genotyping Master Mix**. The genotyping results for each SNP are given for dilutions of 1 ng, 0.1 ng and 0.01 ng (first, second and third row respectively). Genotypes derived from direct sequencing were used as reference for determining concordance. Click here for file

Additional file 3 **Values of calls, concordant genotypes, concordance rate, call rate and genotype concordance in genomic and amplified artificially-degraded DNA**. The results reported are obtained from MDA amplification of high artificially-degraded DNA (180 s). Genotypes derived from direct sequencing were used as reference for determining concordance. Click here for file
