# Supplementary material for: Whole genome amplification and real-time PCR in forensic casework
Source: BMC Genomics. 2009 Apr 14;10:159. doi: 10.1186/1471-2164-10-159 (PMC2675535; doi:10.1186/1471-2164-10-159)
Supplement: Additional file 1 — Values of calls, concordant genotypes, concordance rate, call rate and genotype concordance in genomic and amplified DNA with TaqMan® Universal Master Mix. The genotyping results for each SNP are given for dilutions of 1 ng, 0.1 ng and 0.01 ng (first, second and third row respectively). Genotypes derived from direct sequencing were used as reference for determining concordance. [file 1471-2164-10-159-S1.doc]

| **Table 1.** | **Values of calls, concordant genotypes, concordance rate, call rate and genotype concordance in genomic and amplified DNA with TaqMan® Universal Master Mix.** | | | | | | | | | | |
| --- | --- | --- | --- | --- | --- | --- | --- | --- | --- | --- | --- |
|  | |  | **GENOMIC DNA** | | |  |  | **MDA DNA** | | |  |
|  | | **Runs** | **Calls** | **Concordant genotype** | **Calls rate** | **Concordance rate** | **Runs** | **Calls** | **Concordant genotype** | **Calls rate** | **Concordance rate** |
|  | | 100 | 100 | 100 | 100% | 100% | 100 | 100 | 100 | 100% | 100% |
| **rs1779866** | | 100 | 100 | 100 | 100% | 100% | 100 | 100 | 100 | 100% | 100% |
|  | | 100 | 100 | 100 | 100% | 100% | 100 | 99 | 99 | 99.0% | 100% |
|  | | 100 | 100 | 100 | 100% | 100% | 100 | 100 | 100 | 100% | 100% |
| **rs1922807** | | 100 | 100 | 100 | 100% | 100% | 100 | 100 | 100 | 100% | 100% |
|  | | 100 | 100 | 100 | 100% | 100% | 100 | 100 | 100 | 100% | 100% |
|  | | 100 | 100 | 100 | 100% | 100% | 100 | 100 | 100 | 100% | 100% |
| **rs2278741** | | 100 | 100 | 100 | 100% | 100% | 100 | 100 | 100 | 100% | 100% |
|  | | 100 | 100 | 100 | 100% | 100% | 100 | 99 | 97 | 99.0% | 97.9% |
|  | | 100 | 100 | 100 | 100% | 100% | 100 | 100 | 100 | 100% | 100% |
| **rs2962594** | | 100 | 100 | 100 | 100% | 100% | 100 | 100 | 100 | 100% | 100% |
|  | | 100 | 100 | 100 | 100% | 100% | 100 | 100 | 100 | 100% | 100% |
|  | | 100 | 100 | 100 | 100% | 100% | 100 | 100 | 100 | 100% | 100% |
| **rs905213** | | 100 | 100 | 100 | 100% | 100% | 100 | 100 | 100 | 100% | 100% |
|  | | 100 | 100 | 100 | 100% | 100% | 100 | 100 | 100 | 100% | 100% |
|  | | 100 | 100 | 100 | 100% | 100% | 100 | 100 | 100 | 100% | 100% |
| **rs1075665** | | 100 | 100 | 100 | 100% | 100% | 100 | 100 | 100 | 100% | 100% |
|  | | 100 | 100 | 100 | 100% | 100% | 100 | 100 | 100 | 100% | 100% |
|  | | 100 | 100 | 100 | 100% | 100% | 100 | 100 | 100 | 100% | 100% |
| **rs11242909** | | 100 | 100 | 100 | 100% | 100% | 100 | 100 | 100 | 100% | 100% |
|  | | 100 | 100 | 100 | 100% | 100% | 100 | 98 | 98 | 98.0% | 100% |
|  | | 100 | 100 | 100 | 100% | 100% | 100 | 100 | 100 | 100% | 100% |
| **rs3130315** | | 100 | 98 | 98 | 98.0% | 100% | 100 | 100 | 100 | 100% | 100% |
|  | | 100 | 96 | 96 | 96.0% | 100% | 100 | 100 | 100 | 100% | 100% |
|  | | 100 | 100 | 100 | 100% | 100% | 100 | 100 | 100 | 100% | 100% |
| **rs7740233** | | 100 | 100 | 100 | 100% | 100% | 100 | 100 | 100 | 100% | 100% |
|  | | 100 | 100 | 100 | 100% | 100% | 100 | 100 | 98 | 100% | 98.0% |
|  | | 100 | 100 | 100 | 100% | 100% | 100 | 100 | 100 | 100% | 100% |
| **rs10866988** | | 100 | 98 | 98 | 98.0% | 100% | 100 | 100 | 100 | 100% | 100% |
|  | | 100 | 100 | 100 | 100% | 100% | 100 | 100 | 98 | 100% | 98.0% |
|  | | 100 | 100 | 100 | 100% | 100% | 100 | 100 | 100 | 100% | 100% |
| **rs585070** | | 100 | 94 | 94 | 94.0% | 100% | 100 | 100 | 100 | 100% | 100% |
|  | | 100 | 98 | 98 | 98.0% | 100% | 100 | 98 | 98 | 98.0% | 100% |
|  | | 100 | 100 | 100 | 100% | 100% | 100 | 100 | 100 | 100% | 100% |
| **rs1506981** | | 100 | 100 | 100 | 100% | 100% | 100 | 100 | 100 | 100% | 100% |
|  | | 100 | 86 | 86 | 86.0% | 100% | 100 | 99 | 99 | 99% | 100% |
|  | | 100 | 100 | 100 | 100% | 100% | 100 | 100 | 100 | 100% | 100% |
| **rs1533800** | | 100 | 100 | 100 | 100% | 100% | 100 | 100 | 100 | 100% | 100% |
|  | | 100 | 100 | 100 | 100% | 100% | 100 | 99 | 95 | 99.0% | 95.9% |
|  | | 100 | 100 | 100 | 100% | 100% | 100 | 100 | 100 | 100% | 100% |
| **rs1981752** | | 100 | 100 | 100 | 100% | 100% | 100 | 100 | 99 | 100% | 99% |
|  | | 100 | 100 | 100 | 100% | 100% | 100 | 100 | 100 | 100% | 100% |
|  | | 100 | 100 | 100 | 100% | 100% | 100 | 100 | 100 | 100% | 100% |
| **rs478347** | | 100 | 100 | 100 | 100% | 100% | 100 | 100 | 100 | 100% | 100% |
|  | | 100 | 96 | 96 | 96.0% | 100% | 100 | 100 | 100 | 100% | 100% |
|  | | 100 | 100 | 100 | 100% | 100% | 100 | 100 | 100 | 100% | 100% |
| **rs9562080** | | 100 | 100 | 100 | 100% | 100% | 100 | 100 | 100 | 100% | 100% |
|  | | 100 | 100 | 100 | 100% | 100% | 100 | 100 | 100 | 100% | 100% |
|  | | 100 | 100 | 100 | 100% | 100% | 100 | 100 | 100 | 100% | 100% |
| **rs911621** | | 100 | 100 | 100 | 100% | 100% | 100 | 100 | 100 | 100% | 100% |
|  | | 100 | 100 | 100 | 100% | 100% | 100 | 98 | 98 | 98.0% | 100% |
|  | | 100 | 100 | 100 | 100% | 100% | 100 | 100 | 100 | 100% | 100% |
| **rs999842** | | 100 | 100 | 100 | 100% | 100% | 100 | 100 | 100 | 100% | 100% |
|  | | 100 | 100 | 100 | 100% | 100% | 100 | 96 | 96 | 94.0% | 100% |
|  | | 100 | 100 | 100 | 100% | 100% | 100 | 100 | 100 | 100% | 100% |
| **rs8033863** | | 100 | 100 | 100 | 100% | 100% | 100 | 100 | 100 | 100% | 100% |
|  | | 100 | 100 | 100 | 100% | 100% | 100 | 100 | 100 | 100% | 100% |
|  | | 100 | 100 | 100 | 100% | 100% | 100 | 100 | 100 | 100% | 100% |
| **rs886528** | | 100 | 100 | 100 | 100% | 100% | 100 | 100 | 100 | 100% | 100% |
|  | | 100 | 100 | 100 | 100% | 100% | 100 | 99 | 99 | 99.0% | 100% |
|  | | 100 | 100 | 100 | 100% | 100% | 100 | 100 | 100 | 100% | 100% |
| **rs154659** | | 100 | 100 | 100 | 100% | 100% | 100 | 100 | 100 | 100% | 100% |
|  | | 100 | 100 | 100 | 100% | 100% | 100 | 99 | 97 | 99.0% | 97.9% |
|  | | 100 | 100 | 100 | 100% | 100% | 100 | 100 | 100 | 100% | 100% |
| **rs2317225** | | 100 | 100 | 100 | 100% | 100% | 100 | 100 | 100 | 100% | 100% |
|  | | 100 | 100 | 100 | 100% | 100% | 100 | 100 | 99 | 100% | 99% |
|  | | 100 | 100 | 100 | 100% | 100% | 100 | 100 | 100 | 100% | 100% |
| **rs873289** | | 100 | 96 | 96 | 96.0% | 100% | 100 | 100 | 100 | 100% | 100% |
|  | | 100 | 94 | 94 | 94.0% | 100% | 100 | 100 | 97 | 100% | 97.0% |
|  | | 100 | 100 | 100 | 100% | 100% | 100 | 99 | 99 | 99.0% | 100% |
| **rs11881170** | | 100 | 100 | 100 | 100% | 100% | 100 | 99 | 99 | 99.0% | 100% |
|  | | 100 | 100 | 100 | 100% | 100% | 100 | 98 | 98 | 98.0% | 100% |
|  | | 100 | 100 | 100 | 100% | 100% | 100 | 100 | 100 | 100% | 100% |
| **rs380011** | | 100 | 100 | 100 | 100% | 100% | 100 | 100 | 100 | 100% | 100% |
|  | | 100 | 100 | 100 | 100% | 100% | 100 | 98 | 98 | 98.0% | 100% |
|  | | 100 | 100 | 100 | 100% | 100% | 100 | 100 | 100 | 100% | 100% |
| **rs2267628** | | 100 | 100 | 100 | 100% | 100% | 100 | 100 | 100 | 100% | 100% |
|  | | 100 | 100 | 100 | 100% | 100% | 100 | 100 | 100 | 100% | 100% |
|  | | **2600** | **2600** | **2600** | **100%** | **100%** | **2600** | **2599** | **2599** | **99.961%** | **100%** |
| **Total:** | | **2600** | **2586** | **2586** | **99.461%** | **100%** | **2600** | **2599** | **2598** | **99.961%** | **99.961%** |
|  | | **2600** | **2570** | **2570** | **98.846%** | **100%** | **2600** | **2580** | **2564** | **99.230%** | **99.379%** |

The genotyping results for each SNP are given for dilutions of 1 ng, 0.1 ng and 0.01 ng (first, second and third row respectively). Genotypes derived from direct sequencing were used as reference for determining concordance.
