# Supplementary material for: Whole genome amplification and real-time PCR in forensic casework
Source: BMC Genomics. 2009 Apr 14;10:159. doi: 10.1186/1471-2164-10-159 (PMC2675535; doi:10.1186/1471-2164-10-159)
Supplement: Additional file 2 — Values of calls, concordant genotypes, concordance rate, call rate and genotype concordance in genomic and amplified DNA with TaqMan® Genotyping Master Mix. The genotyping results for each SNP are given for dilutions of 1 ng, 0.1 ng and 0.01 ng (first, second and third row respectively). Genotypes derived from direct sequencing were used as reference for determining concordance. [file 1471-2164-10-159-S2.doc]

| **Table 2.** | **Values of calls, concordant genotypes, concordance rate, call rate and genotype concordance in genomic and amplified DNA with TaqMan® Genotyping l Master Mix.** | | | | | | | | | | |
| --- | --- | --- | --- | --- | --- | --- | --- | --- | --- | --- | --- |
|  | |  | **GENOMIC DNA** | | |  |  | **MDA DNA** | | |  |
|  | | **Runs** | **Calls** | **Concordant genotypes** | **Calls rate** | **Concordance**  **rate** | **Runs** | **Calls** | **Concordant genotypes** | **Calls rate** | **Concordance rate** |
|  | | 100 | 100 | 100 | 100% | 100% | 100 | 100 | 100 | 100% | 100% |
| **rs1779866** | | 100 | 100 | 100 | 100% | 100% | 100 | 100 | 100 | 100% | 100% |
|  | | 100 | 100 | 100 | 100% | 100% | 100 | 100 | 100 | 100% | 100% |
|  | | 100 | 100 | 100 | 100% | 100% | 100 | 100 | 100 | 100% | 100% |
| **rs1922807** | | 100 | 100 | 100 | 100% | 100% | 100 | 100 | 100 | 100% | 100% |
|  | | 100 | 100 | 100 | 100% | 100% | 100 | 100 | 100 | 100% | 100% |
|  | | 100 | 100 | 100 | 100% | 100% | 100 | 100 | 100 | 100% | 100% |
| **rs2278741** | | 100 | 100 | 100 | 100% | 100% | 100 | 100 | 100 | 100% | 100% |
|  | | 100 | 100 | 100 | 100% | 100% | 100 | 100 | 99 | 100% | 99.0% |
|  | | 100 | 100 | 100 | 100% | 100% | 100 | 100 | 100 | 100% | 100% |
| **rs2962594** | | 100 | 100 | 100 | 100% | 100% | 100 | 100 | 100 | 100% | 100% |
|  | | 100 | 100 | 100 | 100% | 100% | 100 | 100 | 100 | 100% | 100% |
|  | | 100 | 100 | 100 | 100% | 100% | 100 | 100 | 100 | 100% | 100% |
| **rs905213** | | 100 | 100 | 100 | 100% | 100% | 100 | 100 | 100 | 100% | 100% |
|  | | 100 | 100 | 100 | 100% | 100% | 100 | 100 | 100 | 100% | 100% |
|  | | 100 | 100 | 100 | 100% | 100% | 100 | 100 | 100 | 100% | 100% |
| **rs1075665** | | 100 | 100 | 100 | 100% | 100% | 100 | 100 | 100 | 100% | 100% |
|  | | 100 | 100 | 100 | 100% | 100% | 100 | 100 | 100 | 100% | 100% |
|  | | 100 | 100 | 100 | 100% | 100% | 100 | 100 | 100 | 100% | 100% |
| **rs11242909** | | 100 | 100 | 100 | 100% | 100% | 100 | 100 | 100 | 100% | 100% |
|  | | 100 | 100 | 100 | 100% | 100% | 100 | 100 | 100 | 100% | 100% |
|  | | 100 | 100 | 100 | 100% | 100% | 100 | 100 | 100 | 100% | 100% |
| **rs3130315** | | 100 | 100 | 100 | 100% | 100% | 100 | 100 | 100 | 100% | 100% |
|  | | 100 | 100 | 100 | 100% | 100% | 100 | 100 | 100 | 100% | 100% |
|  | | 100 | 100 | 100 | 100% | 100% | 100 | 100 | 100 | 100% | 100% |
| **rs7740233** | | 100 | 100 | 100 | 100% | 100% | 100 | 100 | 100 | 100% | 100% |
|  | | 100 | 100 | 100 | 100% | 100% | 100 | 100 | 100 | 100% | 100% |
|  | | 100 | 100 | 100 | 100% | 100% | 100 | 100 | 100 | 100% | 100% |
| **rs10866988** | | 100 | 100 | 100 | 100% | 100% | 100 | 100 | 100 | 100% | 100% |
|  | | 100 | 100 | 100 | 100% | 100% | 100 | 100 | 100 | 100% | 100% |
|  | | 100 | 100 | 100 | 100% | 100% | 100 | 100 | 100 | 100% | 100% |
| **rs585070** | | 100 | 100 | 100 | 100% | 100% | 100 | 100 | 100 | 100% | 100% |
|  | | 100 | 100 | 100 | 100% | 100% | 100 | 99 | 99 | 99.0% | 100% |
|  | | 100 | 100 | 100 | 100% | 100% | 100 | 100 | 100 | 100% | 100% |
| **rs1506981** | | 100 | 100 | 100 | 100% | 100% | 100 | 100 | 100 | 100% | 100% |
|  | | 100 | 100 | 100 | 100% | 100% | 100 | 100 | 100 | 100% | 100% |
|  | | 100 | 100 | 100 | 100% | 100% | 100 | 100 | 100 | 100% | 100% |
| **rs1533800** | | 100 | 100 | 100 | 100% | 100% | 100 | 100 | 100 | 100% | 100% |
|  | | 100 | 100 | 100 | 100% | 100% | 100 | 100 | 100 | 100% | 100% |
|  | | 100 | 100 | 100 | 100% | 100% | 100 | 100 | 100 | 100% | 100% |
| **rs1981752** | | 100 | 100 | 100 | 100% | 100% | 100 | 100 | 100 | 100% | 100% |
|  | | 100 | 100 | 100 | 100% | 100% | 100 | 100 | 100 | 100% | 100% |
|  | | 100 | 100 | 100 | 100% | 100% | 100 | 100 | 100 | 100% | 100% |
| **rs478347** | | 100 | 100 | 100 | 100% | 100% | 100 | 100 | 100 | 100% | 100% |
|  | | 100 | 100 | 100 | 100% | 100% | 100 | 100 | 100 | 100% | 100% |
|  | | 100 | 100 | 100 | 100% | 100% | 100 | 100 | 100 | 100% | 100% |
| **rs9562080** | | 100 | 100 | 100 | 100% | 100% | 100 | 100 | 100 | 100% | 100% |
|  | | 100 | 100 | 100 | 100% | 100% | 100 | 100 | 100 | 100% | 100% |
|  | | 100 | 100 | 100 | 100% | 100% | 100 | 100 | 100 | 100% | 100% |
| **rs911621** | | 100 | 100 | 100 | 100% | 100% | 100 | 100 | 100 | 100% | 100% |
|  | | 100 | 100 | 100 | 100% | 100% | 100 | 99 | 99 | 99.0% | 100% |
|  | | 100 | 100 | 100 | 100% | 100% | 100 | 100 | 100 | 100% | 100% |
| **rs999842** | | 100 | 100 | 100 | 100% | 100% | 100 | 100 | 100 | 100% | 100% |
|  | | 100 | 100 | 100 | 100% | 100% | 100 | 100 | 100 | 100% | 100% |
|  | | 100 | 100 | 100 | 100% | 100% | 100 | 100 | 100 | 100% | 100% |
| **rs8033863** | | 100 | 100 | 100 | 100% | 100% | 100 | 100 | 100 | 100% | 100% |
|  | | 100 | 100 | 100 | 100% | 100% | 100 | 100 | 100 | 100% | 100% |
|  | | 100 | 100 | 100 | 100% | 100% | 100 | 100 | 100 | 100% | 100% |
| **rs886528** | | 100 | 100 | 100 | 100% | 100% | 100 | 100 | 100 | 100% | 100% |
|  | | 100 | 100 | 100 | 100% | 100% | 100 | 100 | 100 | 100% | 100% |
|  | | 100 | 100 | 100 | 100% | 100% | 100 | 100 | 100 | 100% | 100% |
| **rs154659** | | 100 | 100 | 100 | 100% | 100% | 100 | 100 | 100 | 100% | 100% |
|  | | 100 | 100 | 100 | 100% | 100% | 100 | 100 | 100 | 100% | 100% |
|  | | 100 | 100 | 100 | 100% | 100% | 100 | 100 | 100 | 100% | 100% |
| **rs2317225** | | 100 | 100 | 100 | 100% | 100% | 100 | 100 | 100 | 100% | 100% |
|  | | 100 | 100 | 100 | 100% | 100% | 100 | 100 | 100 | 100% | 100% |
|  | | 100 | 100 | 100 | 100% | 100% | 100 | 100 | 100 | 100% | 100% |
| **rs873289** | | 100 | 100 | 100 | 100% | 100% | 100 | 100 | 100 | 100% | 100% |
|  | | 100 | 100 | 100 | 100% | 100% | 100 | 100 | 100 | 100% | 100% |
|  | | 100 | 100 | 100 | 100% | 100% | 100 | 100 | 100 | 100% | 100% |
| **rs11881170** | | 100 | 100 | 100 | 100% | 100% | 100 | 100 | 100 | 100% | 100% |
|  | | 100 | 100 | 100 | 100% | 100% | 100 | 100 | 99 | 100% | 99.0% |
|  | | 100 | 100 | 100 | 100% | 100% | 100 | 100 | 100 | 100% | 100% |
| **rs380011** | | 100 | 100 | 100 | 100% | 100% | 100 | 100 | 100 | 100% | 100% |
|  | | 100 | 100 | 100 | 100% | 100% | 100 | 100 | 100 | 100% | 100% |
|  | | 100 | 100 | 100 | 100% | 100% | 100 | 100 | 100 | 100% | 100% |
| **rs2267628** | | 100 | 100 | 100 | 100% | 100% | 100 | 100 | 100 | 100% | 100% |
|  | | 100 | 100 | 100 | 100% | 100% | 100 | 100 | 100 | 100% | 100% |
|  | | **2600** | **2600** | **2600** | **100%** | **100%** | **2600** | **2600** | **2600** | **100%** | **100%** |
| **Total:** | | **2600** | **2600** | **2600** | **100%** | **100%** | **2600** | **2600** | **2600** | **100%** | **100%** |
|  | | **2600** | **2600** | **2600** | **100%** | **100%** | **2600** | **2598** | **2596** | **99.923%** | **99.923%** |

The genotyping results for each SNP are given for dilutions of 1 ng, 0.1 ng and 0.01 ng (first, second and third row respectively). Genotypes derived from direct sequencing were used as reference for determining concordance
