# Supplementary material for: Whole genome amplification and real-time PCR in forensic casework
Source: BMC Genomics. 2009 Apr 14;10:159. doi: 10.1186/1471-2164-10-159 (PMC2675535; doi:10.1186/1471-2164-10-159)
Supplement: Additional file 3 — Values of calls, concordant genotypes, concordance rate, call rate and genotype concordance in genomic and amplified artificially-degraded DNA. The results reported are obtained from MDA amplification of high artificially-degraded DNA (180 s). Genotypes derived from direct sequencing were used as reference for determining concordance. [file 1471-2164-10-159-S3.doc]

| **Table 3.** | **Values of calls, concordant genotypes, concordance rate, call rate and genotype concordance in genomic and amplified artificially-degraded DNA** | | | | | | | | | | |
| --- | --- | --- | --- | --- | --- | --- | --- | --- | --- | --- | --- |
|  | |  | **GENOMIC DNA** | | |  |  | **MDA DNA** | | |  |
|  | | **Runs** | **Calls** | **Concordant genotype** | **Calls rate** | **Concordance rate** | **Runs** | **Calls** | **Concordant genotype** | **Calls rate** | **Concordance rate** |
| **rs1779866** | | 10 | 9 | 9 | 90.0% | 100% | 10 | 10 | 10 | 100% | 100% |
| **rs1922807** | | 10 | 10 | 10 | 100% | 100% | 10 | 10 | 10 | 100% | 100% |
| **rs2278741** | | 10 | 10 | 10 | 100% | 100% | 10 | 10 | 10 | 100% | 100% |
| **rs2962594** | | 10 | 10 | 10 | 100% | 100% | 10 | 10 | 10 | 100% | 100% |
| **rs905213** | | 10 | 10 | 10 | 100% | 100% | 10 | 10 | 10 | 100% | 100% |
| **rs1075665** | | 10 | 6 | 6 | 60% | 100% | 10 | 10 | 10 | 100% | 100% |
| **rs11242909** | | 10 | 10 | 10 | 100% | 100% | 10 | 10 | 10 | 100% | 100% |
| **rs3130315** | | 10 | 10 | 10 | 100% | 100% | 10 | 10 | 10 | 100% | 100% |
| **rs7740233** | | 10 | 10 | 10 | 100% | 100% | 10 | 10 | 10 | 100% | 100% |
| **rs10866988** | | 10 | 10 | 10 | 100% | 100% | 10 | 10 | 10 | 100% | 100% |
| **rs585070** | | 10 | 7 | 7 | 70% | 100% | 10 | 10 | 10 | 100% | 100% |
| **rs1506981** | | 10 | 10 | 10 | 100% | 100% | 10 | 10 | 10 | 100% | 100% |
| **rs1533800** | | 10 | 10 | 10 | 100% | 100% | 10 | 10 | 10 | 100% | 100% |
| **rs1981752** | | 10 | 10 | 10 | 100% | 100% | 10 | 10 | 10 | 100% | 100% |
| **rs478347** | | 10 | 10 | 10 | 100% | 100% | 10 | 10 | 10 | 100% | 100% |
| **rs9562080** | | 10 | 10 | 10 | 100% | 100% | 10 | 10 | 10 | 100% | 100% |
| **rs911621** | | 10 | 10 | 10 | 100% | 100% | 10 | 10 | 10 | 100% | 100% |
| **rs999842** | | 10 | 10 | 10 | 100% | 100% | 10 | 10 | 10 | 100% | 100% |
| **rs8033863** | | 10 | 10 | 10 | 100% | 100% | 10 | 10 | 10 | 100% | 100% |
| **rs886528** | | 10 | 9 | 9 | 90.0% | 100% | 10 | 10 | 10 | 100% | 100% |
| **rs154659** | | 10 | 10 | 10 | 100% | 100% | 10 | 10 | 10 | 100% | 100% |
| **rs2317225** | | 10 | 10 | 10 | 100% | 100% | 10 | 10 | 10 | 100% | 100% |
| **rs873289** | | 10 | 10 | 10 | 100% | 100% | 10 | 10 | 10 | 100% | 100% |
| **rs11881170** | | 10 | 10 | 10 | 100% | 100% | 10 | 10 | 10 | 100% | 100% |
| **rs380011** | | 10 | 10 | 10 | 100% | 100% | 10 | 10 | 10 | 100% | 100% |
| **rs2267628** | | 10 | 10 | 10 | 100% | 100% | 10 | 10 | 10 | 100% | 100% |
| **Total:** | | **260** | **251** | **251** | **96.53%** | **100%** | **260** | **260** | **260** | **100%** | **100%** |

The results reported are obtained from MDA amplification of high artificially-degraded DNA (180 s). Genotypes derived from direct sequencing were used as reference for determining concordance.
